# Diagnostic, Therapeutic Predictive, and Prognostic Value of Neutrophil Extracellular Traps in Patients With Gastric Adenocarcinoma

**DOI:** 10.3389/fonc.2020.01036

**Published:** 2020-06-26

**Authors:** Yiyin Zhang, Yangyang Hu, Cui Ma, Hua Sun, Xiaoli Wei, Min Li, Wei Wei, Fei Zhang, Feng Yang, Hua Wang, Kangsheng Gu

**Affiliations:** ^1^Department of Oncology, The First Affiliated Hospital of Anhui Medical University, Hefei, China; ^2^Department of Pathology, Basic Medical School of Anhui Medical University, Hefei, China

**Keywords:** stomach neoplasms, neutrophil extracellular traps, biomarker, peripheral blood, prognosis

## Abstract

Neutrophils are a significant population of infiltrated immune cells in the tumor microenvironment. Neutrophil extracellular traps (NETs) are implicated in the biological behavior of many malignant tumors. NETs can be degraded into soluble nucleosomes, leading to the release of fragments containing DNA and granule proteins into the peripheral blood (PB). Using human gastric cancer (GC) biopsies and PB samples, we investigated the specific value of NETs in GC from a clinical perspective. In summary, the formation of NETs was discovered in the tissue microenvironment and PB of GC patients. The amounts of NETs and neutrophil accumulation decreased from tumor tissue to paratumor tissue. In addition, the level of NETs in the PB gradually declined through the following patient populations: advanced disease patients, preoperative patients, postoperative patients, benign disease patients, and healthy controls. The levels of NETs in the plasma and serum were significantly correlated. As a serum biomarker, NETs had a better diagnostic value than carcinoembryonic antigen (CEA) and carbohydrate antigen 19-9 (CA19-9) in GC. The neutrophil count and neutrophil to lymphocyte ratio (NLR) were significantly associated with the level of NETs in the PB. The existence of lymph node metastasis indicated a high level of NETs in the serum. Moreover, the level of NETs in the PB was inversely correlated with short-term efficacy in GC patients who had received advanced first-line treatment. The higher baseline level of NETs in the PB of patients with negative HER2 status was correlated with worse progression-free survival (PFS). And the level of NETs in the PB was a unfavorable independent prognostic factor for PFS in patients with advanced GC who had received first-line treatment. Thus, NETs have novel diagnostic, therapeutic predictive, and prognostic value in GC patients.

## Introduction

Gastric cancer (GC) is one of the most common and aggressive cancers worldwide (1). Currently, the prognosis of GC is still poor. Current biomarkers exhibit poor sensitivity and specificity. It is noteworthy that GC occurrence, progression and prognosis are related to pivotal immune cells in the tumor microenvironment, not just the tumor cells themselves (2). On the other hand, the tumor microenvironment is a dominant force in multidrug resistance (3).

Neutrophils are the most abundant immune cell type in the peripheral blood (PB). In addition, neutrophils are a predominant type of infiltrated immune cell in GC tissue (4). It is well-known that neutrophils are the first line of defense against infections by pathogens. However, neutrophils also play important roles in cancer-related inflammation in both the PB circulation and the tumor microenvironment. For example, the median survival time of GC patients with a low neutrophil to lymphocyte ratio (NLR) in the PB is longer than that of patients with a high NLR (5). Our preliminary study has shown that GC tissues with neutrophil infiltration are related to significantly worse survival than those without neutrophil infiltration (6).

In the inflammatory response, neutrophils play critical roles through three major mechanisms. These processes include phagocytosis, degranulation, and the release of neutrophil extracellular traps (NETs). In particular, NETs are extracellular neutrophil-derived web-like structures (7). NETosis is the process of neutrophil activation. These specific traps constitute a DNA backbone containing histones [such as Circulating histone H3 (H3Cit)] and neutrophil granule proteins [such as Myeloperoxidase (MPO) and neutrophil elastase (NE)]. Initially, the function of NETs was thought to be defense against pathogens. However, now, NETs are implicated in a number of non-infectious inflammatory conditions, such as cancer-related inflammation (8).

The first study evaluating NETs in tumor tissue reported Ewing sarcoma patients. Tumor-associated NETs were discovered in patients who had a poor prognosis (9). Furthermore, NETs have been detected in colon carcinoma and metastatic lymph nodes. From the tumor nest to the distal margin, the content of NETs gradually decreases (10). Not only in tumor tissues but also in the PB, circulating NETs are involved in tumor-induced systemic effects. NETs can be degraded into soluble nucleosomes, leading to the release of fragments containing DNA and granule proteins into the PB (11). A study showed that the count of NETs in lymphoma tissue samples was positively correlated with plasma levels. A high level of NETs was correlated with poor survival (12). Moreover, another study observed that an increased level of postoperative circulating NETs in patients undergoing attempted curative liver resection for metastatic colorectal cancer was related to a reduction in disease-free survival (13). In patients with primary hepatic carcinoma combined with non-alcoholic steatohepatitis, the PB levels of NETs are higher than those in patients with normal liver histology or benign liver disease (14).

However, the correlation between NETs and GC remains almost unknown. Our previous work demonstrated that MPO-positive neutrophils infiltrated GC tissues (6). Therefore, NETs may also exist in the GC microenvironment. In addition, a recent study reported significantly higher PB levels of NETs in 25 advanced esophagogastric adenocarcinoma patients than in 12 patients with local disease (15). Nevertheless, there is currently a lack of large-sample research data.

Hence, this clinical study sought to determine whether NETs may serve as biomarkers in GC patients. Specifically, our aims were as follows: (i) to examine the colocalization between the formation of NETs in the microenvironment of GC tissues and that of para-GC tissues; (ii) to compare the NETs of GC patients between tissues and components of the PB; (iii) to compare NETs in the PB among GC patients, benign gastric disease patients and normal controls; (iv) to assess the correlations between the amount of NETs and clinicopathologic features in GC patients; (v) to evaluate the correlations between the amount of NETs in the PB and short-term efficacy/survival of patients with GC who had received advanced first-line treatment; and (vi) to show the correlation between NETs in the PB and prognosis.

## Materials and Methods

### Patients and Treatment

This study was approved by the ethics committee at Anhui Medical University, and informed consent was obtained from all participants. Samples from a total of 290 gastric adenocarcinoma patients were used at the First Affiliated Hospital of Anhui Medical University between March 2019 and April 2020. These patients did not have acute infectious disease, rheumatic disease or thrombotic disease. Tumor and paratumor tissue samples were obtained from 30 of these patients through surgery. PB samples were acquired from 290 patients with GC (83 patients before surgery, 85 patients from 1 month after surgery and 122 patients from initial diagnosis of advanced disease to before and after first-line treatment), 70 age- and sex-matched benign gastric disease patients and 85 healthy controls. GC TNM stages were defined based on the American Joint Committee on Cancer Staging Manual (8th Edition). Clinicopathologic data, including age, sex, surgery, clinical stage, pathological tumor stage, tumor nerve invasion, vascular tumor emboli, lymph node metastasis, tumor location, differentiation, distant metastasis, and alcohol consumption (the Alcohol Use Disorders Identification Test, 2017 Chinese version, developed by the World Health Organization), were evaluated. Furthermore, through the PB samples, the carcinoembryonic antigen (CEA) level, carbohydrate antigen 19-9 (CA19-9) level, neutrophil count, NLR, clotting time, fibrinogen degradation product (FDP) level, and D-dimer (D-D) level were obtained by reviewing the medical records of patients.

Advanced first-line treatment was administered at the discretion of each patient's physician and with the agreement of the patients who had an initial diagnosis of advanced GC. These patients had to receive at least two cycles of treatment and a follow-up examination. Advanced chemotherapy regimens included platinum/taxane combined with fluorouracil. If the human epidermal growth factor receptor-2 (HER-2) status was positive, trastuzumab was combined with the above chemotherapy. Short-term efficacy was determined in accordance with Response Evaluation Criteria in Solid Tumors (RECIST, version 1.1), including complete response (CR), partial response (PR), stable disease (SD), and progressive disease (PD). CR+PR was applied to compute the objective response rate (ORR), and CR+PR+SD was used to calculate the disease control rate (DCR). Progression-free survival (PFS) was calculated from the time of diagnosis to disease progression or the last follow-up evaluation. In this study, the follow-up time ended in April 25, 2020.

### Immunofluorescence

NET identification in tissue samples was performed by immunofluorescence staining. An anti-NE antibody (MAB91671) was purchased from R&D Systems (Minneapolis, USA) (1:50 dilution). An anti-H3Cit antibody (ab5103) was obtained from Abcam (Cambridge, UK) (1:50 dilution). The NE/H3Cit pair was researched in paraffin-embedded, 3-μm-thick sections. The slides were incubated with the primary antibodies at 4°C overnight after blocking with goat serum. Then, the sections were incubated with secondary antibodies (Alexa Fluor 488, green; and Alexa Fluor 647, red) (Abcam, Cambridge, UK) for 1 h at room temperature. DAPI was used for nuclear staining (ZSGB Biotech, Beijing, China). Finally, the slides were analyzed with a confocal laser scanning microscope (TCS-SP5; Leica, Wetzlar, Germany). For each specimen, the position of tumor and paratumor tissue was determined according to the results of H&E staining. Next, the average numbers of NE and H3Cit double-positive cells of tumor and paratumor tissues were calculated by two researchers counting five random 630x microscopic fields, respectively.

### Immunohistochemistry

Briefly, slides were baked, dewaxed, and rehydrated. Then, the sections were boiled for antigen retrieval, followed by blocking endogenous peroxidase activity with hydrogen peroxide. Next, the sections were incubated with the primary anti-NE antibody overnight at 4°C. Subsequently, the sections were incubated with a secondary antibody (Elivision super HRP IHC Kit; Maixin, Fujian, China) for 30 min at room temperature. For visualization of proteins, the sections were incubated with diaminobenzidine (Maixin, Fujian, China). Finally, the slides were counterstained with hematoxylin, dehydrated and mounted. The average number of NE-positive cells infiltrating the GC microenvironment was counted in five random 200x microscopic fields by two investigators.

### PB Sample Processing

The time nodes of PB acquisition were arranged as follows. PB samples of advanced patients were collected before the initial treatment and when evaluating short-term outcomes. PB samples of preoperative patients were obtained at first diagnosis before operation. Moreover, PB samples of postoperative patients were got from 1 month after surgery before adjuvant therapy to avoid errors caused by postoperative stress. Neutrophils, serum and plasma were isolated from PB samples by centrifugation. The serum and plasma were used for measuring NE-DNA complexes. Neutrophils were cultured in RPMI 1640 base medium. Then, 1 μl of 1 mM phorbol myristate acetate (PMA) or phosphate-buffered solution (PBS) was added to the neutrophil culture medium to obtain supernatants that were used as positive or negative controls.

### Quantification of NETs in the PB

NE attached to nucleosomes was considered to indicate NET formation in the serum and plasma of PB samples. NE-DNA complexes were identified by using capture ELISA (11). Then, 5 μg/ml anti-NE antibody (MAB91672) (R&D Systems; Minneapolis, USA) was used to coat 96-well microtiter plates (50 μl per well) overnight at 4°C. After blocking in 1% BSA, 40 μl of serum or plasma was added to the wells with a peroxidase-labeled anti-DNA antibody (11774425001) (component No. 2 of the commercial cell death detection ELISA kit; Roche, Mannheim, Germany). After an incubation for 2 h with shaking (320 rpm) at room temperature, the wells were washed three times with PBS. A peroxidase substrate from a kit (ABTS) (11774425001) (Roche, Mannheim, Germany) was added to the wells. The optical density (OD) was measured at a 405-nm wavelength using a microplate reader (Synergy HTX; Bio Tek, USA) after 40 min of incubation at 37°C in the dark. The OD value was used to reflect the quantification of NETs in the PB.

### Statistical Analysis

All statistical analyses were performed using GraphPad Prism software version 6.0 (GraphPad, La Jolla, USA) or SPSS version 17.0 (SPSS, Chicago, USA). The significance of NETs and neutrophil accumulation in GC and para-GC tissue samples was assessed by the Wilcoxon test. The correlation in NET formation between PB and tumor tissue samples was tested by the Pearson correlation coefficient or Spearman non-parametric analysis. The significance of NETs in PB samples between groups of participants was determined using the Mann-Whitney *U*-test. The diagnostic capability of NETs was evaluated using a receiver operating characteristic (ROC) curve. The χ^2^ test was used to evaluate the associations between the level of NETs and existing clinicopathological factors. The levels of NETs before and after advanced first-line treatment were compared by the Wilcoxon test. The Kaplan-Meier method and log-rank test were utilized to evaluate survival data. Multivariate analysis was performed by Cox proportional hazards regression modeling. *P* < 0.05 were considered significant.

## Results

### Clinicopathological Characteristics of Participants

The characteristics of the participants are presented in Table 1. Among them, 103 patients with advanced disease received first-line treatment. Short-term efficacy could be assessed in 76 patients. Up to the last follow-up date, Disease progression was recorded for 53 patients. The time range for follow-up evaluations in all advanced patients was 1.4–10.9 months. Moreover, the median PFS time was 5.5 months in the 76 patients.

**Table 1:** Clinicopathological characteristics of GC patients and controls.

**Characteristics**	**122 patients with advanced disease**		**83 patients with a preoperative status**		**85 patients with a postoperative status**		**70 patients with benign gastric disease**		**85 healthy volunteers**	
	**Number**	***N*%**	**Number**	***N*%**	**Number**	***N*%**	**Number**	***N*%**	**Number**	***N*%**
**Age, Years**										
>=65	63	51.7	48	57.8	37	43.5	16	22.9	26	30.6
<65	59	48.3	35	42.2	48	56.5	54	77.1	59	69.4
**Sex**										
Male	97	79.5	61	73.5	61	71.8	38	54.3	49	57.6
Female	25	20.5	22	26.5	24	28.2	32	45.7	36	42.4
**Clinical Stage**										
I + II	0	0	52	62.7	37	43.5	-	-	-	-
III + IV	122	100.0	31	37.3	48	56.5	-	-	-	-
**Pathological Tumor Stage**										
T1 + T2	-	-	33	39.8	17	20.0	-	-	-	-
T3 + T4	-	-	50	60.2	68	80.0	-	-	-	-
**Tumor Nerve Invasion**										
Yes	-	-	51	61.4	56	65.9	-	-	-	-
No	-	-	32	38.6	29	34.1	-	-	-	-
**Vascular Tumor Emboli**										
Yes	-	-	54	65.1	52	61.2	-	-	-	-
No	-	-	29	34.9	33	38.8	-	-	-	-
**Lymph Node Metastasis**										
Yes	74	60.7	43	51.8	66	77.6	-	-	-	-
No	48	39.3	40	48.2	19	22.4	-	-	-	-
**Lung Metastasis**										
Yes	13	10.7	-	-	-	-	-	-	-	-
No	109	89.3	-	-	-	-	-	-	-	-
**Hepatic Metastasis**										
Yes	39	32.0	-	-	-	-	-	-	-	-
No	83	68.0	-	-	-	-	-	-	-	-
**Peritoneal Metastasis**										
Yes	53	43.4	-	-	-	-	-	-	-	-
No	69	56.6	-	-	-	-	-	-	-	-
**Tumor Location**										
Proximal	60	49.2	46	55.4	48	56.5	-	-	-	-
Distal	62	50.8	37	44.6	37	43.5	-	-	-	-
**Differentiation**										
Poor	65	53.3	30	36.1	48	56.5	-	-	-	-
Moderate and well	57	46.7	53	63.9	37	43.5	-	-	-	-
**Alcohol Consumption**										
Hazardous or harmful	39	32.0	10	12.0	20	23.5	14	20.0	-	-
Abstinence or low risk	83	68.0	73	88.0	65	76.5	56	80.0	-	-

*-, not covered*.

### NETs in the Tumor Microenvironment of GC

**Figure 1:**
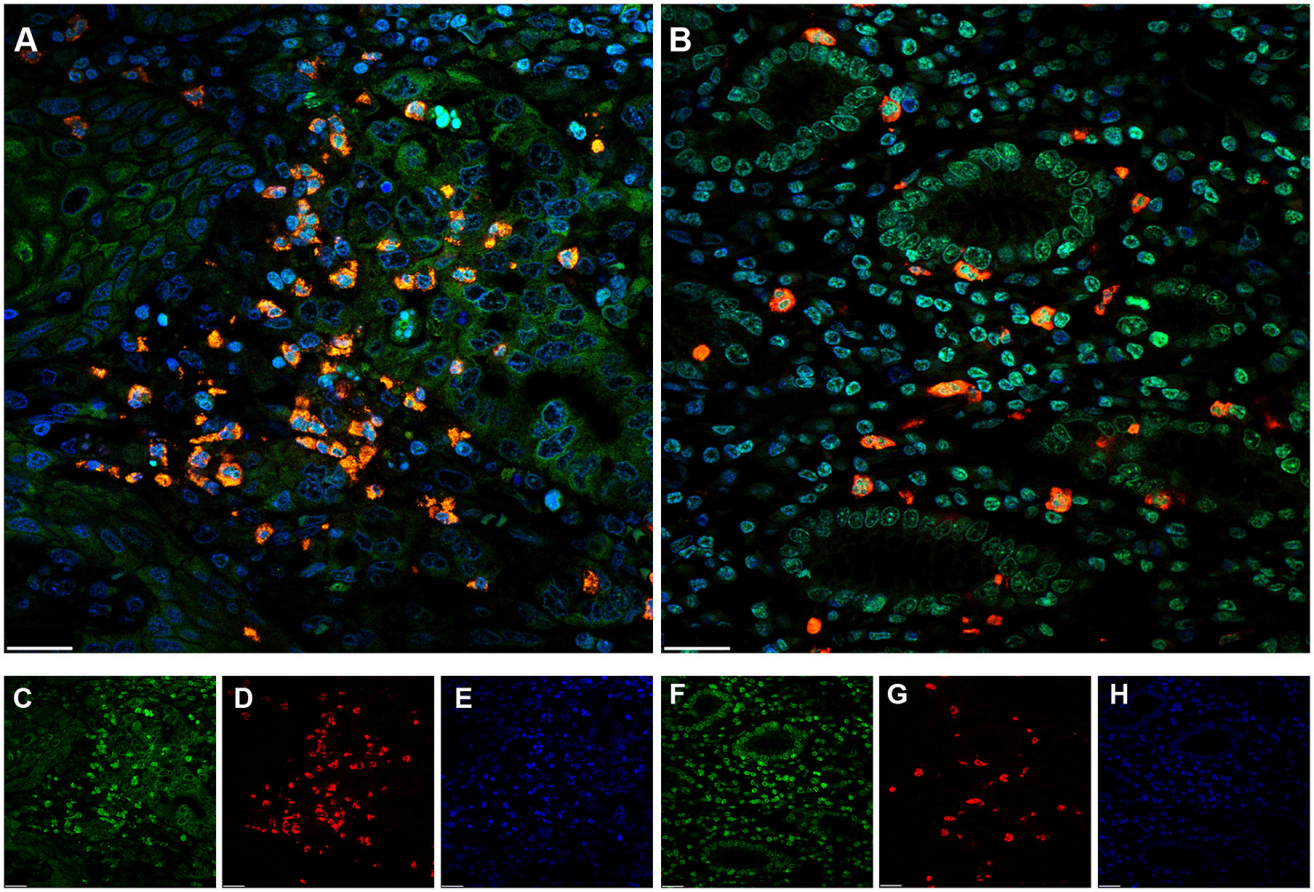
Immunofluorescence staining of neutrophil extracellular traps (NETs) in gastric cancer (GC). **(A)** Colocalization of neutrophil elastase (NE) and Circulating histone H3 (H3Cit) in the microenvironment of tumor tissue. **(B)** Colocalization of NE and H3Cit in the microenvironment of paratumor tissue. **(C)** H3Cit. **(D)** NE. **(E)** DAPI. **(F)** H3Cit. **(G)** NE. **(H)** DAPI. Scale bars: 25 μm.

**Figure 2:**
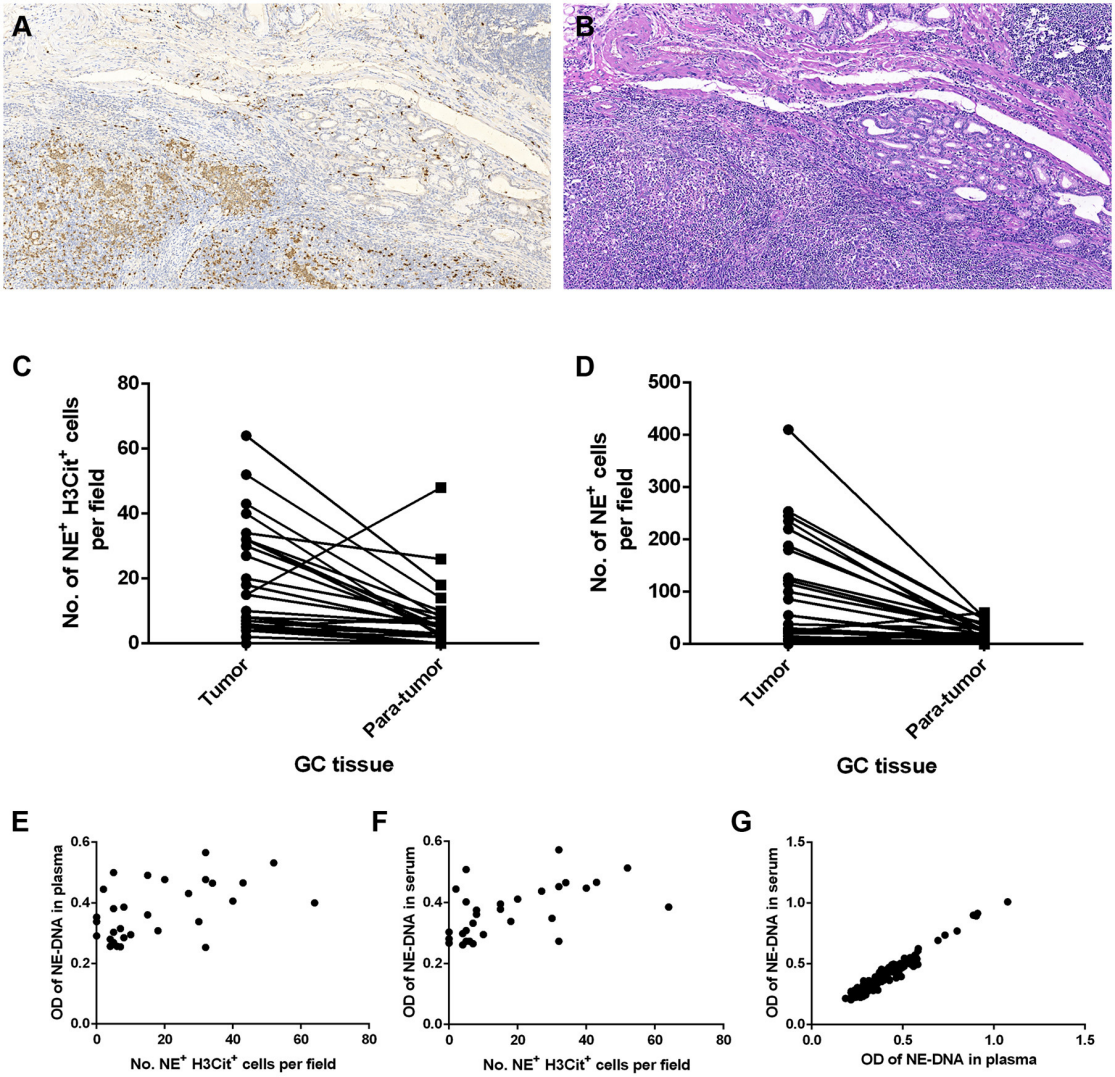
Neutrophil accumulation and neutrophil extracellular traps (NETs) in gastric cancer (GC) tissue and peripheral blood (PB) components collected from patients. **(A)** Immunohistochemical staining for neutrophil elastase (NE) in GC. **(B)** H&E staining of GC tissue. **(C)** Reduced median numbers of NET formations per field in paratumor tissue samples compared with tumor tissue samples (*P* < 0.001). **(D)** Decreased neutrophil accumulation in paratumor tissue samples compared with tumor tissue samples (*P* < 0.001). **(E,F)** Statistically significant correlations between the formation of NETs in tumor tissue samples and that in the components of PB samples (**E**: tissue and plasma, *r* = 0.456, *P* = 0.011; **F**: tissue and serum, *r* = 0.580, *P* = 0.001). **(G)** Statistically significant correlation between the formation of NETs in plasma and that in serum (*r* = 0.973, *P* < 0.001). Original magnification: 100x **(A,B)**.

To investigate whether NETs can be detected in the tumor microenvironment of GC, we performed immunofluorescence staining on samples from 30 patients with GC. Intriguingly, NETs were observed in the tumor microenvironment (Figures 1A–H), as colocalization of NE with H3Cit was observed by confocal microscopy. Furthermore, neutrophil accumulation was discovered in the tumor tissues through immunohistochemical staining for NE (Figures 2A,B). Compared with those in the tumor tissue samples, the median numbers of NET formations per field in the paratumor tissue samples were decreased significantly (*P* < 0.001) (Figure 2C). Meanwhile, neutrophil accumulation was diminished in the paratumor tissue samples from the same patients (*P* < 0.001) (Figure 2D).

### Association of the Levels of NETs in GC Tissue Samples With Those in PB Samples

The correlation between the levels of NETs in tumor tissue samples and those in PB samples was determined using the Pearson coefficient or Spearman non-parametric analysis. The association was statistically significant regarding the NET levels in GC tissue and PB samples from the same patient (plasma: Spearman *r* = 0.456, *P* = 0.011; serum: Spearman *r* = 0.580, *P* = 0.001) (Figures 2E,F). In addition, we compared the levels of NETs between the plasma and serum of GC patients. There was also a statistically significant correlation between the NET levels in the plasma and serum in 283 PB samples from GC patients (Pearson coefficient *r* = 0.973, *P* < 0.001) (Figure 2G).

### Comparing NETs in the PB Among Participants

**Figure 3:**
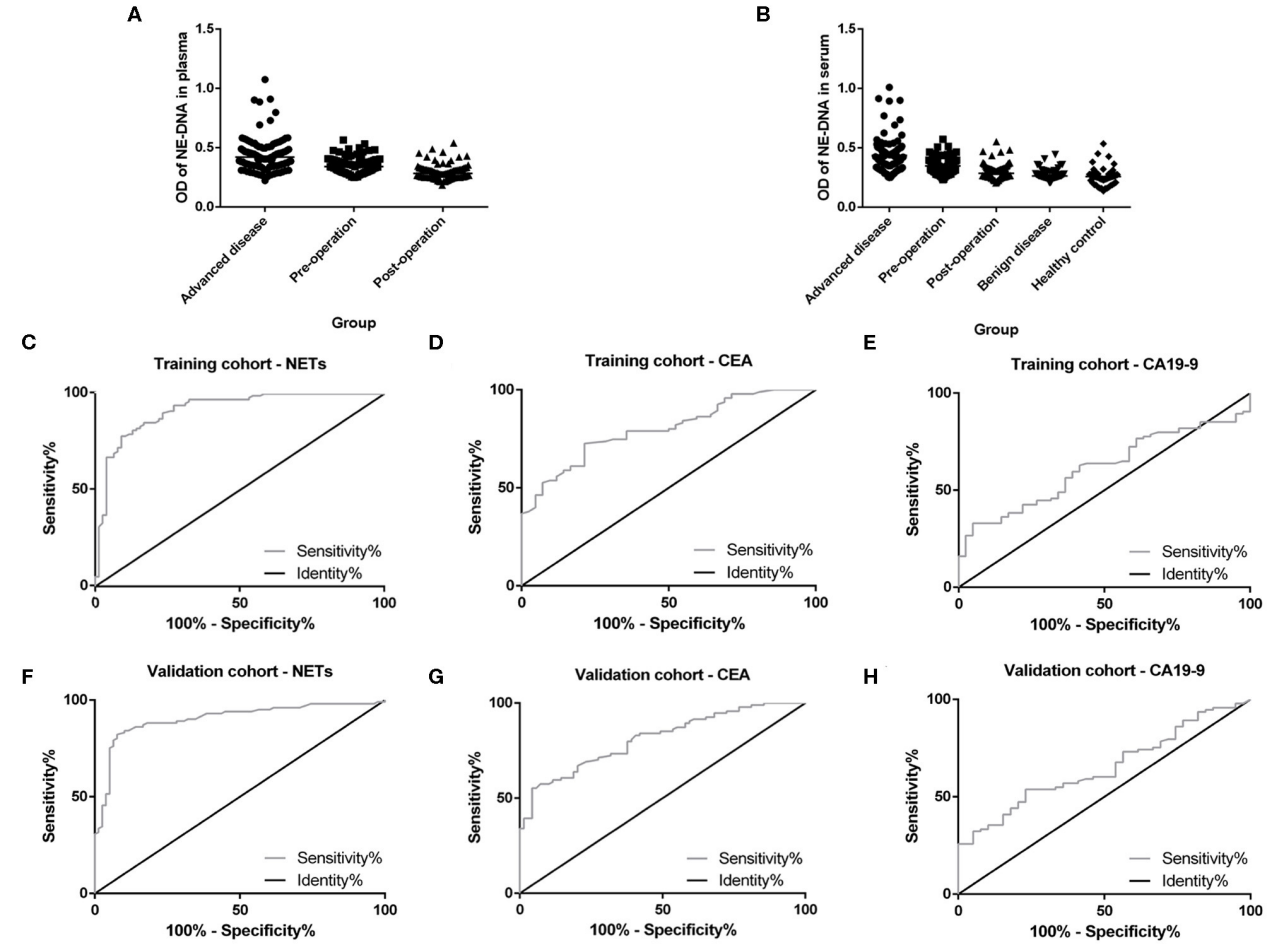
Neutrophil extracellular traps (NETs) in the peripheral blood (PB) of participants and the diagnostic capacity in gastric cancer (GC). **(A)** Gradual decline in the levels of NETs in the plasma from advanced disease patients to postoperative patients. **(B)** Gradual decline in levels of NETs in the serum from advanced disease patients to controls. **(C,F)** Diagnostic capacity of NETs in the training cohort (AUC = 0.916; **C**) and the validation cohort (AUC=0.903; **F**). **(D,G)** Diagnostic capacity of carcinoembryonic antigen (CEA) in the training cohort (AUC = 0.799; **D**) and the validation cohort (AUC = 0.812; **G**). **(E,H)** Diagnostic capacity of carbohydrate antigen 19-9 (CA19-9) in the training cohort (AUC = 0.614) and validation cohort (AUC = 0.650; **H**).

Plasma and serum samples were obtained from participants in each group. Among the groups, 122, 83, and 85 plasma specimens were collected from GC patients with advanced disease, GC patients before surgery and GC patients after surgery, respectively. On the other hand, 119, 82, and 82 serum samples were obtained from the same groups of participants described above. In addition, 70 and 85 serum samples were collected from patients with benign gastric disease and healthy controls, respectively. We discovered that the median levels of NETs in the plasma were 0.422, 0.341, and 0.283 in the patients with advanced disease (Group A), the preoperative patients (Group B), and the postoperative patients (Group C), respectively (Table 2). The levels of NETs gradually declined from advanced disease patients to patients after surgery (Figure 3A). Similarly, in the serum, the median amounts of NETs were 0.435, 0.349, 0.286, 0.264, and 0.258 in the patients with advanced disease (Group A), preoperative patients (Group B), postoperative patients (Group C), gastric benign disease patients (Group D), and healthy volunteers (Group E), respectively. Hence, the levels of NETs also decreased gradually from metastatic disease to benign disease and healthy tissue (Table 2, Figure 3B). However, the levels of NETs in the benign gastric disease patients were not significantly higher than those in the healthy volunteers (Table 2).

**Table 2:** Levels of NETs in PB samples from groups of participants.

**Samples**	**Statistics**	**A vs. B**	**A vs. C**	**A vs. D**	**A vs. E**	**B vs. C**	**B vs. D**	**B vs. E**	**C vs. D**	**C vs. E**	**D vs. E**
Plasma	*U*-value	2,845	1,312	-	-	1,734	-	-	-	-	-
	*P*	<0.001	<0.001	-	-	<0.001	-	-	-	-	-
Serum	*U*-value	2,569	1,212	492	573	1,741	884	888	2,063	2,049	2,436
	*P*	<0.001	<0.001	<0.001	<0.001	<0.001	<0.001	<0.001	0.003	<0.001	0.052

*-, not covered; A, patients with advanced disease; B, patients with a preoperative status; C, patients with a postoperative status; D, patients with benign gastric disease; and E, healthy volunteers*.

### Diagnostic Value About NETs in GC Patients

To evaluate the diagnostic value of NETs in the serum of PB samples, NET levels were compared with CEA and CA19-9 levels by using ROC curves. We chose GC patients with an initial diagnosis without any treatment (201 cases) and benign disease patients or healthy volunteers (155 cases). And then, all these participants were divided into two groups randomly, including 177 participants (100 GC patients and 77 controls) as the training cohort and 179 participants (101 GC patients and 78 controls) as the validation cohort. There were no significant relationships about clinicopathological characteristics of participants between training cohort and validation cohort (Table 3). In the training cohort, the areas under the curve (AUCs) for NETs, CEA, and CA19-9 were 0.916, 0.799, and 0.614, respectively (Figures 3C–E). In addition, in the validation cohort, the AUCs for NETs, CEA, and CA19-9 were 0.903, 0.812, and 0.650, respectively (Figures 3F–H). Consistent with the training cohort, NETs had a better diagnostic performance than CEA or CA19-9 about GC in the validation cohort (Table 4).

**Table 3:** The relationships about clinicopathological characteristics of participants between training cohort and validation cohort.

**Characteristics**	**GC patients**		**Controls**	
	**Statistic**	***P*-value**	**Statistic**	***P*-value**
Age	0.312	0.755	1.275	0.204
Sex	0.088	0.766	0.516	0.472
Clinical stage	1.196	0.274	-	-
Tumor location	0.605	0.437	-	-
Differentiation	0.048	0.827	-	-

*-, not covered*.

**Table 4:** Diagnostic performance of NETs in the serum of GC patients.

**Biomarkers**	**Training cohort**				**Validation cohort**			
	**Sensitivity**	**Specificity**	**AUC**	**Youden index value**	**Sensitivity**	**Specificity**	**AUC**	**Youden index value**
NETs	78.00%	90.91%	0.916	0.689	82.18%	92.31%	0.903	0.745
CEA	72.63%	78.57%	0.799	0.512	55.32%	95.65%	0.812	0.510
CA19-9	32.98%	95.12%	0.614	0.281	53.76%	76.92%	0.650	0.307

### Correlations Between NETs and Clinicopathological Features in GC Patients

**Figure 4:**
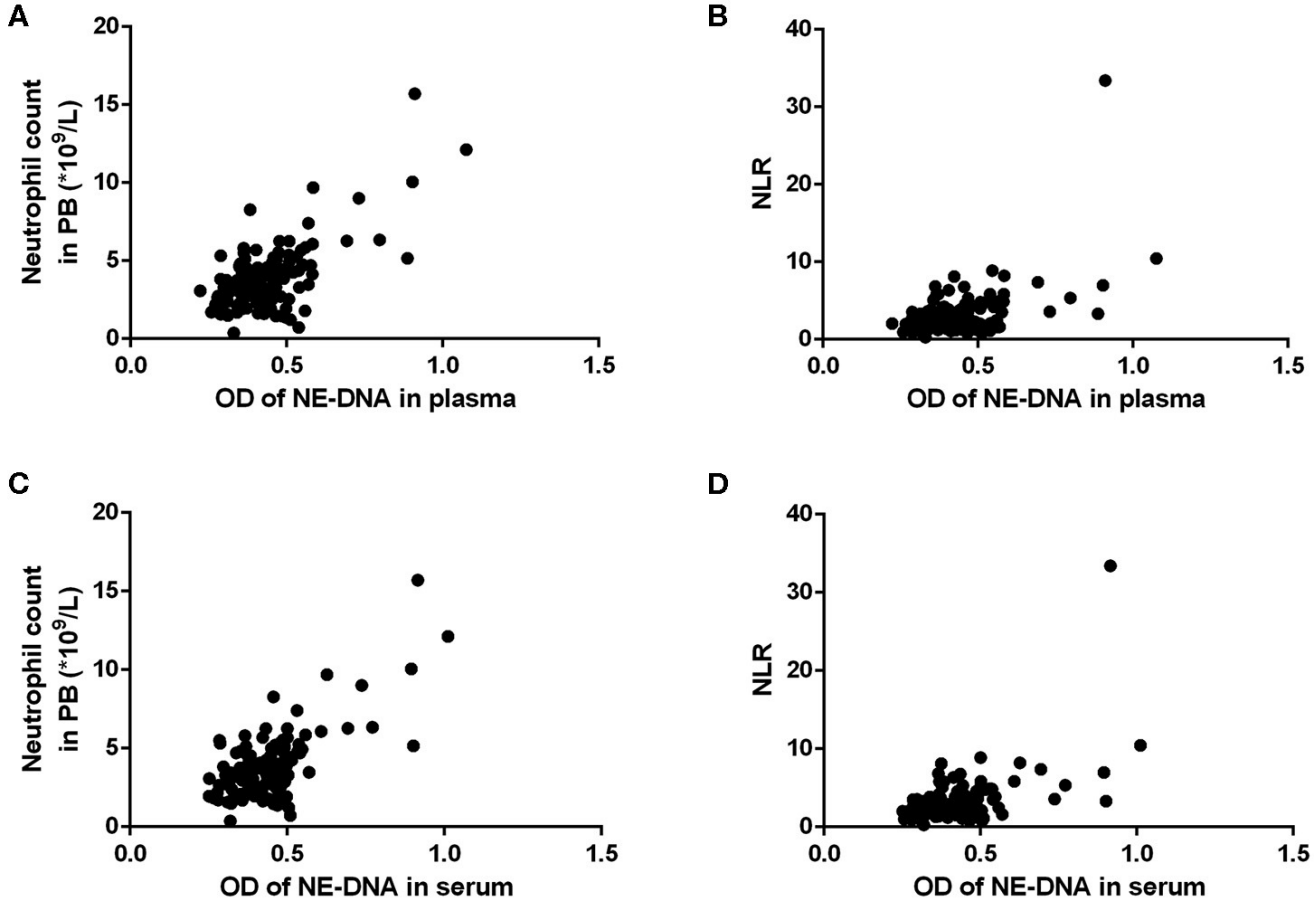
The correlations between the levels of neutrophil extracellular traps (NETs) and the neutrophil count/neutrophil to lymphocyte ratio (NLR) in the peripheral blood (PB) of patients with advanced gastric cancer (GC). **(A,B)** There were statistically significant correlations between the levels of NETs in the plasma and the neutrophil count/NLR (**A**: neutrophil count, *r* = 0.661, *P* < 0.001; **B**: NLR, *r* = 0.527, *P* < 0.001). **(C,D)** There were statistically significant correlations between the levels of NETs in the serum and the neutrophil count/NLR (**C**: neutrophil count, *r* = 0.676, *P* < 0.001; **D**: NLR, *r* = 0.524, *P* < 0.001).

In GC patients with advanced disease, as the optimal cut-off values for high and low levels, the median NET values in the plasma and serum were 0.422 (0.223–1.075) and 0.435 (0.251–1.011), respectively. The neutrophil count and NLR in the PB were significantly associated with the levels of NETs in both the plasma and the serum (Table 5, Figure 4). Moreover, there was an extremely weak correlation between NETs and CA19-9 in the serum in advanced GC patients (Table 5).

**Table 5:** The relationships between the levels of NETs in the PB and clinicopathological features in advanced GC patients.

**Characteristics**	**Plasma**			**Serum**		
		**Statistic**	***P*-value**		**Statistic**	***P*-value**
Age	χ^2^	0.525	0.469	χ^2^	3.700	0.054
Sex		2.465	0.116		0.753	0.386
Distant lymph node metastasis		0.137	0.711		0.005	0.942
Lung metastasis		0.086	0.769		0.069	0.793
Liver metastasis		0.339	0.560		2.097	0.148
Peritoneal metastasis		0.834	0.361		0.434	0.510
Tumor location		0.131	0.717		1.416	0.234
Differentiation		0.296	0.586		0.074	0.786
Alcohol consumption		0.339	0.560		0.019	0.891
CEA	*r*	0.050	0.596	*r*	0.068	0.475
CA19-9		0.185	0.051		0.198	0.038
Neutrophil count in the PB		0.661	<0.001		0.676	<0.001
Lymphocyte count in the PB		0.065	0.477		0.022	0.811
NLR		0.527	<0.001		0.524	<0.001
Blood clotting time		0.028	0.761		<0.001	0.998
FDP		0.122	0.205		0.129	0.186
D-D		0.168	0.079		0.159	0.102

On the other hand, in pre- and postoperative patients, the median NET values in the plasma and serum were 0.311 (0.185–0.566) and 0.316 (0.205–0.572), respectively, which were used as optimal cut-off values for high and low levels. Among these patients, the levels of NETs in PB samples were influenced by whether the patients had undergone surgery (Table 6, Figures 3A,B). Furthermore, the serum levels of NETs had a statistically significant association with the lymph node metastasis status (Table 6). It seemed that the existence of lymph node metastasis indicated high levels of NETs. In addition, there were weak correlations between NETs and some hematological indicators in the PB, such as CEA, CA19-9, the neutrophil count and the NLR (Table 6).

**Table 6:** The relationships between the levels of NETs in the PB and clinicopathological features in GC patients who underwent surgery.

**Characteristics**	**Plasma**			**Serum**		
		**Statistic**	***P*-value**		**Statistic**	***P*-value**
Age	χ^2^	0.214	0.643	χ^2^	0.390	0.532
Sex		<0.001	>0.999		0.276	0.600
Pre- and postoperation		20.027	<0.001		16.488	<0.001
Clinical stage		1.171	0.279		0.612	0.434
Pathological tumor stage		0.456	0.500		2.357	0.125
Tumor nerve invasion		1.261	0.261		2.628	0.105
Vascular tumor emboli		0.409	0.522		0.421	0.517
Lymph node metastasis		0.653	0.419		6.829	0.009
Tumor location		<0.001	>0.999		0.098	0.754
Differentiation		0.383	0.536		1.204	0.273
Alcohol consumption		0.162	0.687		0.689	0.406
CEA	*r*	0.260	0.001	*r*	0.287	<0.001
CA19-9		0.302	<0.001		0.314	<0.001
Neutrophil count in the PB		0.362	<0.001		0.343	<0.001
Lymphocyte count in the PB		0.007	0.931		0.011	0.887
NLR		0.223	0.004		0.207	0.008
Blood clotting time		0.032	0.688		0.028	0.729
FDP		0.022	0.798		0.015	0.866
D-D		0.045	0.598		0.026	0.767

Besides, in terms of the tumor tissue, NET values for high and low levels were nine double-positive cells per field. However, there were no relationships between levels of NETs in the tumor tissue from GC patients and clinicopathologic factors (Table 7).

**Table 7:** The relationships between the levels of NETs in the tumor tissue and clinicopathological features in GC patients.

**Characteristics**	**Tumor tissue**		
		**Statistic**	***P*-value**
Age	χ^2^	1.200	0.273
Sex		2.540	0.111
Clinical stage		0.600	0.439
Pathological tumor stage		0.536	0.464
Tumor nerve invasion		0.556	0.456
Vascular tumor emboli		<0.001	>0.999
Lymph node metastasis		0.133	0.715
Tumor location		0.635	0.426
Differentiation		0.136	0.713
Alcohol consumption		0.288	0.591
CEA	*r*	0.217	0.257
CA19-9		0.063	0.744
Neutrophil count in the PB		0.265	0.157
Lymphocyte count in the PB		0.146	0.442
NLR		0.162	0.392
Blood clotting time		0.288	0.129
FDP		0.246	0.236
D-D		0.261	0.208

### Efficacy of First-Line Treatment According to the Levels of NETs in PB Samples

**Figure 5:**
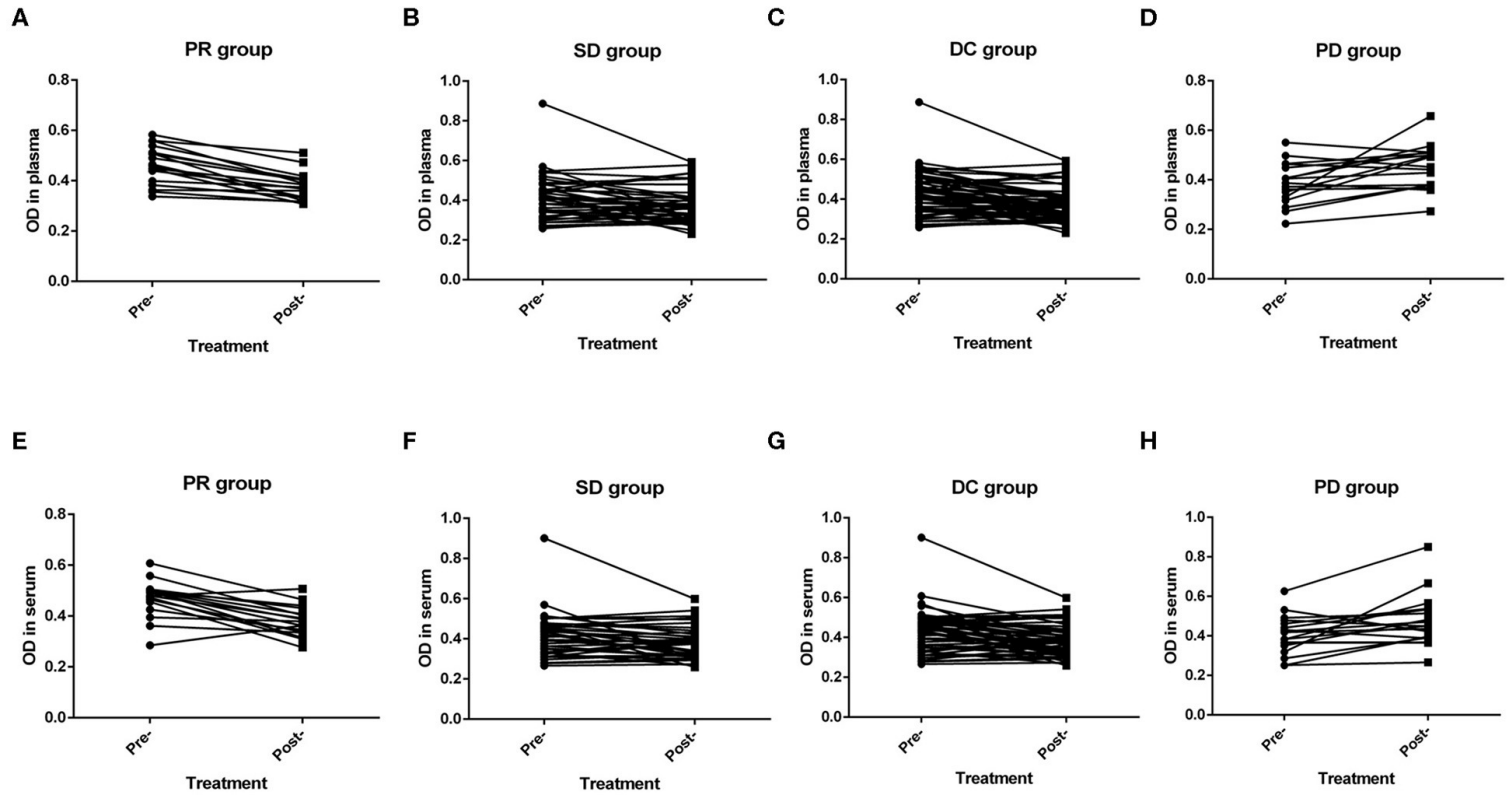
Efficacy of advanced first-line treatment according to the levels of neutrophil extracellular traps (NETs) in the peripheral blood (PB) of patients with gastric cancer (GC). **(A,E)** The levels of NETs before treatment were significantly higher than those after treatment in both the plasma (*P* < 0.001; **A**) and the serum (*P* < 0.001; **E**) in the partial response (PR) group. **(B,F)** The levels of NETs before treatment were significantly higher than those after treatment in both the plasma (*P* = 0.047; **B**) and the serum (*P* = 0.019; **F**) in the stable disease (SD) group. **(C,G)** The levels of NETs before treatment were significantly higher than those after treatment in both the plasma (*P* < 0.001; **C**) and the serum (*P* < 0.001; **G**) in the disease control (DC) group. **(D,H)** The levels of NETs before treatment were significantly lower than those after treatment in both the plasma (*P* = 0.008; **D**) and the serum (*P* = 0.009; **H**) in the progressive disease (PD) group.

As previously mentioned, short-term efficacy could be evaluated in 76 patients with advanced disease. Plasma samples were not obtained from two patients after treatment, and serum samples were not obtained from three patients before treatment. No patients achieved a CR. 18, 39 and 19 patients achieved short-term PR, SD, and PD, respectively. Moreover, the ORR and DCR were 23.7% and 75.0%, respectively. In the PR group, the levels of NETs before first-line treatment were significantly higher than those after two cycles of treatment in both the plasma (*P* < 0.001) and the serum (*P* < 0.001) (Figures 5A,E). Furthermore, in the SD group, the levels of NETs before and after first-line therapy also had similar significant differences in the plasma (*P* = 0.047) and serum (*P* = 0.019) (Figures 5B,F). Overall, in the disease control (DC) group, the levels of NETs in the pretreatment patients were higher than those in the posttreatment patients in both the plasma (*P* < 0.001) and the serum (*P* < 0.001) (Figures 5C,G). In contrast, the levels of NETs before treatment were significantly lower than those after first-line therapy in the plasma (*P* = 0.008) and serum (*P* = 0.009) in the PD group (Figures 5D,H).

### Prognostic Significance of NETs in the PB

**Figure 6:**
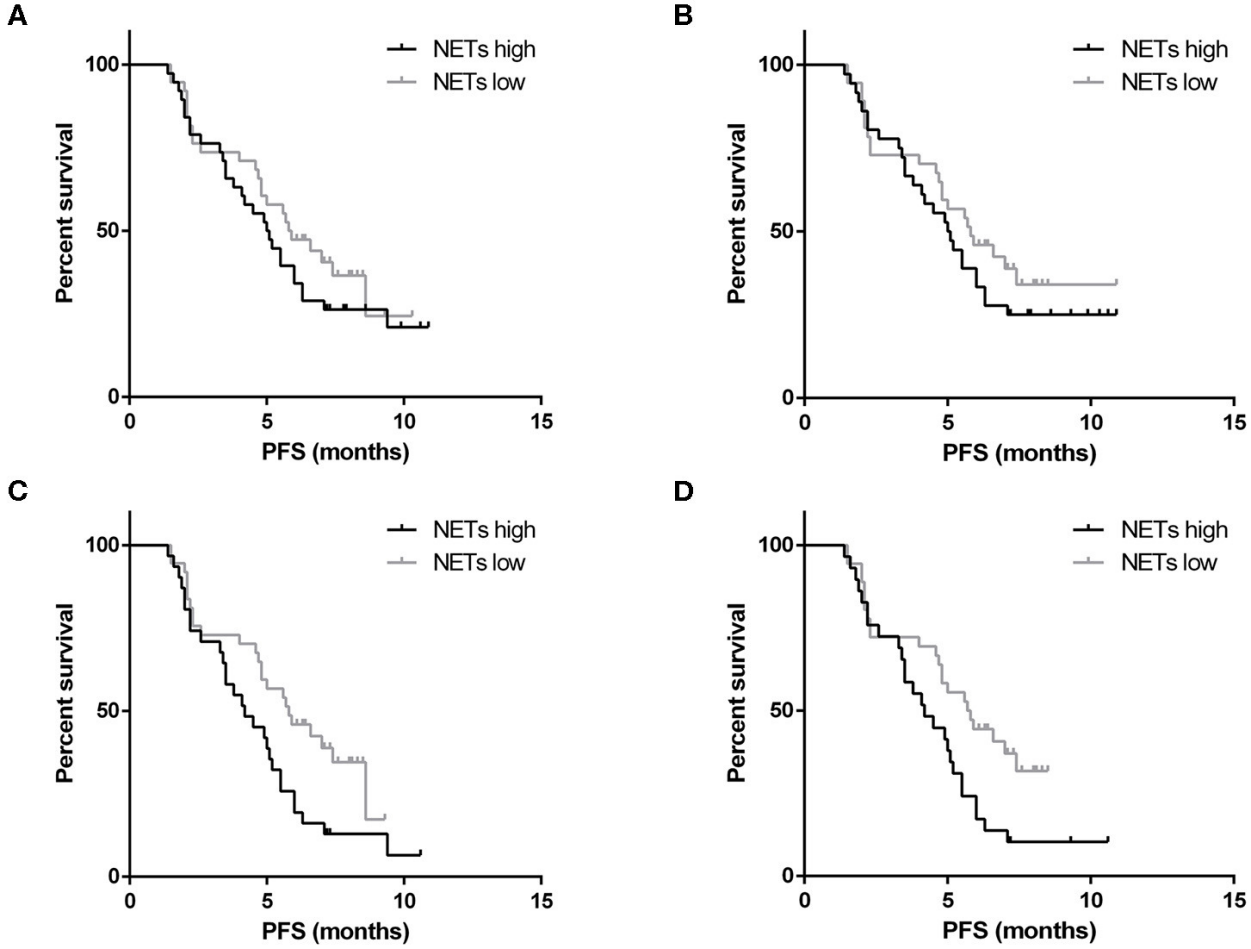
Survival analysis for neutrophil extracellular traps (NETs). There was no correlation between the baseline levels of NETs in the plasma **(A)** or serum **(B)** and the progression-free survival (PFS) of patients with gastric cancer (GC) who had received advanced first-line treatment. In the subgroup analysis, the higher baseline level of NETs in the plasma **(C)** or serum **(D)** of patients with negative HER2 status was correlated with worse PFS.

Disease progression was recorded for 53 patients out of 76 patients who had received advanced first-line treatment. These patients were divided into two groups based on the median baseline levels of NETs in the PB. The cut-off values for the OD were 0.414 and 0.434 in the plasma and serum, respectively. In terms of PFS, the patients with low levels of NETs in the PB were not significantly different from those with high levels of NETs (plasma: 5.9 vs. 5.1 months, respectively, *P* = 0.323; serum: 5.8 vs. 5.1 months, respectively, *P* = 0.368) (Figures 6A,B). In particular, eight patients with positive HER2 status received chemotherapy which combined with Trastuzumab. Among them, disease progression occurred in only one patient. Therefore, subgroup survival was analyzed for 68 patients with negative HER2 status. It revealed that higher levels of NETs were associated with remarkably worse PFS (plasma: 4.2 vs. 5.8 months, *P* = 0.031; serum: 4.2 vs. 5.8 months, *P* = 0.022) (Figures 6C,D). In addition, Cox proportional hazard regression analysis showed that the level of NETs in the PB was a unfavorable independent prognostic factor for PFS in patients with advanced GC who had received first-line treatment (Table 8). Conversely, targeted therapy against HER2 was a favorable independent prognostic factor for PFS in patients with advanced GC who had received first-line treatment (Table 8).

**Table 8:** Multivariate analysis of factors according to PFS in GC patients who had received advanced first-line treatment.

**Factors**	**Plasma**			**Serum**		
	**HR**	**95% CI**	***P*-value**	**HR**	**95% CI**	***P*-value**
NETs	1.891	1.045–3.422	0.035	2.030	1.093–3.769	0.025
Sex	0.742	0.385–1.431	0.373	0.701	0.356–1.380	0.304
Age	1.404	0.751–2.625	0.287	1.614	0.858–3.036	0.138
Distant lymph node metastasis	1.330	0.718–2.464	0.365	1.244	0.671–2.308	0.488
Lung or liver metastasis	0.850	0.467–1.546	0.593	0.913	0.481–1.734	0.781
Peritoneal metastasis	1.099	0.586–2.059	0.769	0.999	0.507–1.967	0.997
Tumor location	0.748	0.417–1.343	0.331	0.630	0.339–1.170	0.143
Differentiation	0.934	0.521–1.671	0.817	1.010	0.543–1.881	0.974
Targeted therapy against HER2	0.057	0.008–0.439	0.006	0.066	0.009–0.505	0.009

## Discussion

NETs have been suggested to contribute to pathogenesis by promoting tumor proliferation (10, 12), metastasis (8, 13, 16) and thrombosis (17, 18) in a variety of tumors. In this study, the formation of NETs was first discovered in the tissue microenvironment of GC. Then, the amounts of NETs and neutrophil accumulation were found to decrease from tumor tissue to paratumor tissue. The levels of NETs in PB samples from GC patients were higher than those in PB samples from gastric benign disease patients and healthy controls, suggesting that neutrophils and released NETs play important roles in the carcinogenesis and tumor development of GC.

For utilizing NETs as a tumor biomarker, the simplicity, repeatability, and accuracy of the detection method for the formation of NETs are particularly significant. Interestingly, NETs are degraded into soluble fragments containing DNA and granule proteins in the PB (11). The granule protein-DNA complexes in the PB were considered to indirectly reflect NETs in the tumor microenvironment in this GC study and previous lymphoma research (12). Furthermore, the plasma and serum are the main components of the PB. In previous studies, NET detection by plasma and serum had been reported in a variety of diseases. For example, the use of plasma included autoimmune small-vessel vasculitis, colorectal cancer and hepatocellular cancer (11, 13, 14). Besides, the use of serum contained lymphoma, esophageal adenocarcinoma, and lung adenocarcinoma (12, 15). Compared with the serum, the plasma contains coagulation factors and fibrinogen. Because of the positive correlation between NETosis and the tumor-promotive effects of hypercoagulation (19), it is important to compare the levels of NETs between the plasma and serum to find the most appropriate component of the PB to use for detecting NETs. In this study, the levels of NETs were confirmed to be highly correlated between the plasma and serum. In addition, the levels of NETs in the plasma and serum of 283 samples from GC patients were not associated with blood coagulation function. This was the first study to compare two components of the PB of cancer patients for the detection of NET formation. However, a previous study suggested that NETs were positively correlated with blood coagulation function in GC patients (17). Notably, that study included only 48 patients with GC. Therefore, we confirm that both the plasma and the serum can be used for NET detection based on our results from 283 samples from GC patients.

In this study, the levels of NETs in PB samples were found to taper from advanced patients to controls. We speculate that this specifically decreasing trend in NET formation is correlated with the tumor burden of GC patients. In particular, patients at 1 month after surgery were compared with preoperative patients in this research. Some studies have reported that surgical stress induced by liver ischemia-reperfusion injury results in widespread NET formation after liver resection (13, 20). Another study also clarified that NET production was elevated for a short time after colorectal cancer resection (21). The interference of the postoperative inflammatory response on NET content must be avoided.

In addition, the diagnostic value of NETs in the serum of GC patients is noteworthy. At present, CEA and CA19-9 are well-known GC biomarkers in the PB. However, the sensitivity and specificity of these serum biomarkers are still limited. Based on our results, NETs had a higher sensitivity, larger AUC and higher Youden index value than CEA and CA19-9 in the training cohort. Furthermore, the above results were verified by the validation cohort. We discovered that diagnostic accuracy was enhanced up to around 0.700. These findings indicated a better diagnostic ability for NETs than the regular biomarkers, such as CEA and CA19-9.

In terms of clinicopathological features, we determined that the levels of NETs were significantly correlated with the neutrophil count and NLR in the PB samples of advanced patients. However, in patients pre- and postoperation, this correlation was weak. This suggests that neutrophil aggregation is the basis of NET production. Surgery may interfere with the outcome of this correlation. On the other hand, there were no relationships between levels of NETs in the tumor tissue and neutrophil count/NLR in the PB. It may be related to the small tissue sample size. In addition, it seemed that the existence of lymph node metastasis indicated high levels of NETs in the serum. In many malignancies, NETs have been suggested to promote tumor metastasis. In mouse experiments, NETs produced during inflammation can awaken dormant cancer cells via key NET-associated proteases, such as NE (22). Another study confirmed that NETs were present in the metastatic lymph nodes of colon carcinoma patients but absent in the normal lymph nodes of the same patients (10). Moreover, NETs can be detected in the omentum of women with early-stage ovarian cancer, and blockade of NETs can prevent omental metastasis (16). Thus, our study obtained similar results in GC from the perspective of clinical patients.

It is worth noting that the presence of NETs increases tumor growth in lung carcinoma and melanoma cell lines (23). As a key component of NETs, NE can induce proliferation in human and mouse adenocarcinoma cell lines. NE deficiency reduces the tumor burden in a mouse lung cancer model (24). In our research, the NE-DNA complex was used to represent the formation of NETs in the PB. We discovered that the amount of the NE-DNA complex gradually declined as the tumor burden decreased. On the other hand, some advanced patients who had received first-line therapy were included in our study. We discovered that the levels of NETs in PB samples were inversely associated with short-term efficacy. Specifically, the levels of NETs were decreased after therapy in GC patients who achieved objective remission or disease control. However, the levels of NETs increased significantly as the disease progressed. In terms of survival and prognosis, according to ToGA trial, Trastuzumab in combination with chemotherapy can prolong the PFS up to 6.7 months compared with 5.5 months in the chemotherapy alone group (25). In our research, Trastuzumab in combination with chemotherapy interfered with the role of baseline levels of NETs in influencing survival in patients with advanced GC. Thus, through the subgroup analysis, we found that higher levels of NETs in the PB were associated with remarkably worse PFS in patients with negative HER2 status. At last, we clarified that the level of NETs in the PB was a unfavorable independent prognostic factor for PFS in patients with advanced GC who had received first-line treatment.

## Conclusions

In conclusion, the formation of NETs was discovered in the tissue microenvironment and PB of GC patients. The amounts of NETs and neutrophil accumulation decreased from tumor tissue to paratumor tissue. In addition, the level of NETs in the PB gradually declined through the following patient populations: advanced disease patients, preoperative patients, postoperative patients, benign disease patients, and healthy controls. In the same patient, there were significant correlations related to not only the amount of NETs in the tumor tissue and the level of NETs in the PB but also the levels of NETs in the plasma and serum. As a serum biomarker, NETs had better diagnostic value than CEA and CA19-9 in GC. The neutrophil count and NLR were significantly associated with the level of NETs in the PB. The existence of lymph node metastasis indicated a high level of NETs in the serum. Moreover, the level of NETs in the PB was inversely correlated with short-term efficacy in GC patients who had received advanced first-line treatment. In addition, the higher baseline level of NETs in the PB of patients with negative HER2 status was correlated with worse PFS. The level of NETs in the PB was a unfavorable independent prognostic factor for PFS in patients with advanced GC who had received first-line treatment. Our results suggest that NETs have novel diagnostic, therapeutic predictive and prognostic value in GC patients.

## Data Availability Statement

All datasets generated for this study are included in the article/supplementary material.

## Ethics Statement

The studies involving human participants were reviewed and approved by the ethics committee of Anhui Medical University. The patients/participants provided their written informed consent to participate in this study.

## Author Contributions

YZ, HW, and KG conceived the hypothesis and study design. YH, CM, and HS collected PB samples. YZ and FY obtained tissue samples. YH, YZ, FY, and XW performed NET quantification through ELISA, immunohistochemistry and immunofluorescence. YZ implemented the statistical analysis. YZ and HW wrote the manuscript. KG, ML, WW, FZ, and YZ managed clinical participants and collected clinicopathological data. All authors approved the final manuscript.

## Conflict of Interest

The authors declare that the research was conducted in the absence of any commercial or financial relationships that could be construed as a potential conflict of interest.

## Abbreviations

NETs: neutrophil extracellular traps
PB: peripheral blood
GC: gastric cancer
CEA: carcinoembryonic antigen
CA19-9: carbohydrate antigen 19-9
NLR: neutrophil to lymphocyte ratio
H3Cit: circulating histone H3
MPO: myeloperoxidase
NE: neutrophil elastase
FDP: fibrinogen degradation product
D-D: D-dimer
HER-2: human epidermal growth factor receptor-2
RECIST: Response Evaluation Criteria in Solid Tumors
CR: complete response
PR: partial response
SD: stable disease
PD: progressive disease
ORR: objective response rate
DCR: disease control rate
PFS: progression-free survival
PMA: phorbol myristate acetate
PBS: phosphate-buffered solution
OD: optical density
ROC: receiver operating characteristic.
